# Selenocystamine improves protein accumulation in chloroplasts of eukaryotic green algae

**DOI:** 10.1186/s13568-015-0126-3

**Published:** 2015-07-03

**Authors:** Livia S Ferreira-Camargo, Miller Tran, Joris Beld, Michael D Burkart, Stephen P Mayfield

**Affiliations:** Division of Biological Sciences, UC San Diego, 9500 Gilman Drive, Bonner Hall, La Jolla, CA 92093-0368 USA; CAPES Foundation, Ministry of Education of Brazil, Brasília, DF 70040-020 Brazil; Department of Chemistry and Biochemistry, UC San Diego, La Jolla, CA 92093-0358 USA

**Keywords:** *Chlamydomonas reinhardtii*, Selenocystamine, Chloroplast, Protein expression, Recombinant protein

## Abstract

Eukaryotic green algae have become an increasingly popular platform for recombinant proteins production. In particular, *Chlamydomonas reinhardtii*, has garnered increased attention for having the necessary biochemical machinery to produce vaccines, human antibodies and next generation cancer targeting immunotoxins. While it has been shown that chloroplasts contain chaperones, peptidyl prolylisomerases and protein disulfide isomerases that facilitate these complex proteins folding and assembly, little has been done to determine which processes serve as rate-limiting steps for protein accumulation. In other expression systems, as *Escherichia coli*, Chinese hamster ovary cells, and insect cells, recombinant protein accumulation can be hampered by cell’s inability to fold the target polypeptide into the native state, resulting in aggregation and degradation. To determine if chloroplasts’ ability to oxidize proteins that require disulfide bonds into a stable conformation is a rate-limiting step of protein accumulation, three recombinant strains, each expressing a different recombinant protein, were analyzed. These recombinant proteins included fluorescent GFP, a reporter containing no disulfide bonds; *Gaussia princeps* luciferase, a luminescent reporter containing disulfide bonds; and an immunotoxin, an antibody-fusion protein containing disulfide bonds. Each strain was analyzed for its ability to accumulate proteins when supplemented with selenocystamine, a small molecule capable of catalyzing the formation of disulfide bonds. Selenocystamine supplementation led to an increase in luciferase and immunotoxin but not GFP accumulation. These results demonstrated that selenocystamine can increase the accumulation of proteins containing disulfide bonds and suggests that a rate-limiting step in chloroplast protein accumulation is the disulfide bonds formation in recombinant proteins native structure.

## Introduction

The advent of recombinant DNA technologies and the ability to transform microbial organisms with synthetic DNA has revolutionized the pharmaceutical industry (Walsh 2014). As a first demonstration, plasmid DNA encoding insulin chain A and B was introduced into *Escherichia coli* and shown to be capable of using the bacterial machinery to produce recombinant insulin for use in patients, albeit at poor yield (Johnson 1983). Since that time, many expression platforms have been developed to harness the unique characteristics of each protein. In instances where post-translational modifications are required, the expression systems of choice are Chinese hamster ovary (CHO) cells, insect cells, or the methylotrophic yeast, *Pichia pastoris* (Ahmad et al. 2014; Kim et al. 2012; Vrljic et al. 2011). When relatively large quantities of simple proteins lacking post-translational modifications are required, *E. coli* is the system of choice (Rosano and Ceccarelli 2014). Since their introduction, each expression system has been examined in detail to determine which factors facilitate protein folding and also to identify the rate-limiting steps of protein production (Sato and Inaba 2012). Once identified, it has been possible to modify the host organism or growth parameters to overcome these rate-limiting steps in order to increase recombinant protein accumulation (Lilie et al. 1994; Sato and Inaba 2012; Horwich et al. 1999).

Recently, eukaryotic green microalgae have been explored as a potential protein production platform. Algae offer attractive production features, including photosynthetic growth (Franklin and Mayfield 2004), ease of genetic manipulations (Grossman 2000), and unique biochemical compartments (Tran et al. 2009). These features allow green algae to produce complex heterologous proteins at a fraction of the cost of traditional protein expression platforms (Franklin and Mayfield 2004). Additionally, many green algae are edible, opening up the possibility of orally delivering bioactive proteins and removing cumbersome and costly downstream purifications associated with other expression systems (Barrera et al. 2014; Gregory et al. 2012). In particular, *Chlamydomonas reinhardtii* has had a full repertoire of genetic tools developed that allow for integration of foreign genes into the mitochondrial, nuclear and chloroplast genomes (Popescu and Lee 2007; Specht et al. 2010). *C. reinhardtii* has also been used to demonstrate the ability of chloroplasts to facilitate the production of full-length human antibodies (Tran et al. 2013b), industrial enzymes (Rasala et al. 2012), and vaccine molecules (Gregory et al. 2012). In some instances, *C. reinhardtii* chloroplasts were capable of accumulating large quantities of recombinant proteins (Manuell et al. 2007). However, when producing more complex proteins, such as full-length human antibodies and immunotoxins, the accumulation levels are relatively low, typically below 1% of total soluble protein (TSP) (Tran et al. 2013b). Although chloroplasts have the machinery to produce and assemble these complex proteins with multiple disulfide bonds (Tran et al. 2013a, b), little has been done to determine which steps of complex protein accumulation are rate-limiting.

*Chlamydomonas reinhardtii* chloroplasts contain chaperones (Schroda 2004), peptidyl propylisomerases (PPIases) (Breiman et al. 1992) and protein disulfide isomerases (PDIs) (Levitan et al. 2005) that are each responsible for catalyzing an important step in the accumulation of complex disulfide bond-containing proteins (Tran et al. 2013b). When disulfide bonds of heterologous proteins are formed incorrectly, aggregation can occur, which ultimately leads to degradation of the mis-folded polypeptide to prevent damage to the host expression organism (Sato and Inaba 2012; Schroder 2008). In *C. reinhardtii*, the PDIs could serve dual functions both as translational activators (Kim and Mayfield 1997) and as enzymes responsible for catalyzing the formation of disulfide bonds (Wilkinson and Gilbert 2004; Levitan et al. 2005). This dual responsibility of PDIs can potentially limit the capacity of *C. reinhardtii* to fold, assemble and accumulate complex proteins containing large numbers of disulfide bonds. To determine if the formation of disulfide bonds is a rate-limiting step in the accumulation of heterologous proteins in *C. reinhardtii* chloroplasts, we used the ability of small molecule diselenides to catalyze oxidative protein folding to interrogate protein accumulation in the algal chloroplast. Previously, it was shown that these small molecule diselenides were able to assist in oxidative protein folding in vitro and in *E. coli* (Beld et al. 2010). Although diselenide bonds are intrinsically stronger than disulfide bonds, the folding energy of the protein upon formation of disulfide bonds, is sufficient to break small molecule diselenide bonds. The formed free selenols are efficient disulfide shuffling reagents and the presence of oxygen recycles quickly the diselenides, and thus these reagents are catalytic. In vivo, it remains the question with which small molecule and protein thiols and disulfides these diselenide reagents interact (Beld et al. 2007; Hondal et al. 2013; Nauser et al. 2012). *E. coli* does not harbor PDI but relies on a separate oxidase DsbA and isomerase DsbC to introduce and reshuffle proteinogenic disulfide bonds. In a DsbA-knockout background, selenocystamine proved to be an especially efficient catalyst of oxidative protein folding. Here we applied a similar technique to shine light on the ability of *C. reinhardtii* chloroplasts to accumulate proteins containing disulfides.

For this study, we used recombinant strain of algae expressing *Gaussiaprinceps* luciferase that require the formation of disulfide bonds for biological activity (Goerke et al. 2008; Tran et al. 2013b), and we also expressed green fluorescent protein (GFP), which does not contain or require disulfide bonds for activity (Prisco et al. 2005). Both recombinant strains were grown in the presence or absence of selenocystamine. Each strain was carefully monitored and analyzed to determine if selenocystamine could increase recombinant protein expression. To test another protein with potential for commercial application, recombinant strain expressing an immunotoxin (complex therapeutic protein containing disulfide bonds), was also grown in the absence or presence of cystamine or selenocystamine at 2 µM. These results helped decipher the rate-limiting steps of protein accumulation *C. reinhardtii* chloroplast, and by these findings, we will be able to target improvements in the algal expression platform that can lead to the development of a robust microalgal expression platform.

## Materials and methods

### *Chlamydomonas reinhardtii* strains

**Figure 1:**
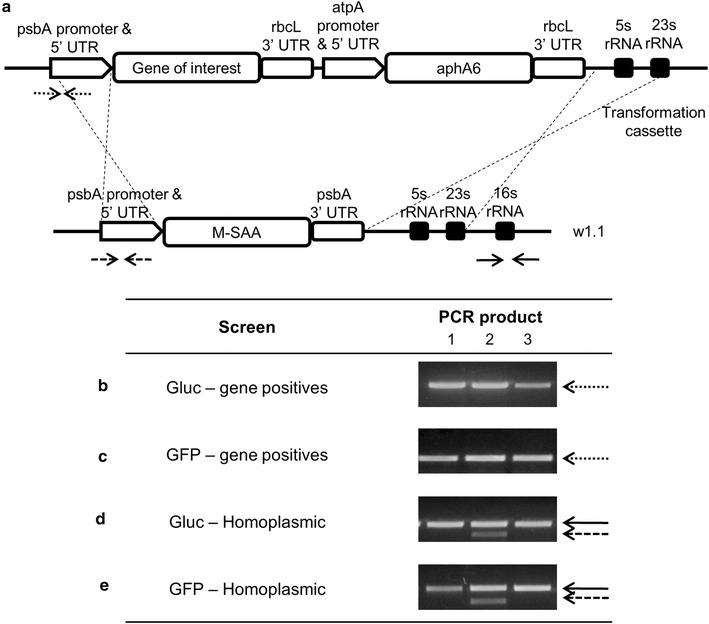
DNA gene constructs, site of integration and identification of transformants in *Chlamydomonas reinhardtii* chloroplast. **a** Transformation vector containing the genes of interest: Gluc (Luciferase from *Gaussia princeps*), or GFP (Green Fluorescent Protein from *Aequorea vcitoria*), or Immunotoxin (αCD22CH23PE40 (Tran et al. 2013b)). Regions of the chloroplast genome were positioned at both ends of the transformation vector to allow homologous integration of the whole transformation cassette into the chloroplast genome (w1.1). **b**, **c** Colony PCR amplification to confirm the presence of recombinant genes of interest, Gluc—Luciferase from *Gaussia princeps* (**b**) or GFP—Green Fluorescent Protein (**c**). **b**, **c** *Lanes 1*, *2* and *3* show three different gene positive colonies (*dotted arrow*). **d**, **e** Colony PCR amplification to check homoplasmicity by using primers from the genome to verify the absence of the second band (*dashed arrow*), which indicates the replacement of SAA by our recombinant genes. The *black arrow* shows the amplified control PCR product. **d**, **e** *Lanes 1* and *3* show homoplasmic colonies, and *lane 2* a control. All the *arrows* (*dotted*, *dashed* and *black*) are also marked (**a**) to indicate the gene amplified regions.

*Chlamydomonas reinhardtii* strain, termed w1.1, is a genetically modified, non-photosynthetic expressing serum amyloid A (SAA) in the psbA site (Manuell et al. 2007). This strain was transformed to obtain the recombinant strains used in this paper and as a control for all the cultivations. The chloroplasts were transformed such that the psbA site was replaced with genes encoding for GFP or luciferase (Gluc) (Figure 1a), generating the respective strains: CC-5117 psbA::pGFP mt+ and CC-5118 psbA::pGluc mt+. The recombinant strain expressing immunotoxin (αCD22HCH23PE40) was previously engineered using the same vector (Figure 1a) (Tran et al. 2013b).

### Plasmid construction

All plasmids were constructed to be transformed into *C. reinhardtii* chloroplasts, and for this reason, all genes were codon optimized to contain adenine and uracil nucleotides in the third position, favoring codons with guanine and cytosine (Nakamura et al. 1999). Two different plasmids were constructed—one encoding GFP from *Aequorea victoria* (pGFP—deposit number at Addgene: 64904) and one encoding Gluc, which contains the luciferase gene from *Gaussia princeps* (pGluc).

Both genes of interest were designed with restriction site *Nde*I at the 5′ end and *Xba*I at the 3′ end, immediately outside of the coding region to facilitate subsequent cloning. Genes were ligated into the psbA transformation vector (Tran and Mayfield 2010), which contains a kanamycin gene (aphA6) as selection marker (Figure 1a).

### *Chlamydomonas reinhardtii* strain transformation

*Chlamydomonas reinhardtii* w1.1 strain was grown in tris-acetate-phosphate (TAP) (Gorman and Levine 1965) liquid medium at 23°C, on rotary shaker set at 100 rpm, under constant light intensity (100 µmol photons m^−2^ s^−1^) provided by fluorescent lamps over 3 days, to a cell concentration between 8 × 10^5^ and 2 × 10^6^ cells mL^−1^. Cells were harvested by centrifugation (2,500 rpm/1,200*g*) and about 1.0 × 10^7^ cells were plated on TAP agar containing 100 µg mL^−1^ of kanamycin. After drying, these cells were transformed by particle bombardment (Boynton et al. 1988). Briefly, 550 nm diameter gold particles (S550d Seashell Technologies, San Diego) covered with 10 µg of plasmid DNA were shot using the gun at a distance of 4 cm at 375 psi.

After 1 week, transformed colonies selected on TAP agar plates containing 100 µg mL^−1^ of kanamycin were patched onto TAP plates containing 150 µg mL^−1^ of kanamycin. Screening for the presence of genes was done by colony PCR using cell lysate (95°C during 10 min) and promoter-specific (psbA) forward primers and gene-specific reverse primers (Figure 1b, c). Homoplasmicity was analyzed by PCR, using the following primers: 5′-GCTTGAATTTATAAATTAAAATATTTTTAC-3′ and 5′-TTCTCTAGCGTTACTGATTACTTTA-3′ for verification of SAA loss at psbA site (w1.1) and primers 5′-CCGAACTGAGGTTGGGTTTA-3′ and 5′-GGGGGAGCGAATAGGATTAG-3′ for verification of the coding region 16S rRNA presence as positive control (Figure 1d, e).

### Oxidative molecules supplementation experiments

Selenocystamine and cystamine, diselenide and disulfide molecules, respectively, were fed to *Chlamydomonas reinhardtii* cultures. Selenocystamine was synthesized following a published procedure (Krief et al. 2004), dissolved in 10 mM HCl, aliquoted, and stored at −20°C.

Inoculum for both recombinant strains was grown in TAP liquid medium from TAP agar, under light conditions (100 µmol photons m^−2^ s^−1^) on a rotary shaker (100 rpm), at 23°C over 3 days, when log phase was achieved. Cells were inoculated in a 250 mL Erlenmeyer flask containing 100 mL liquid TAP using an initial cell concentration of 5 × 10^4^ cells mL^−1^, and these were kept in the dark for 96 h. Afterward, flasks were placed under light conditions (100 µmol photons m^−2^ s^−1^), and this was considered time 0 h of cultivation, when selenocystamine was added. Experiments were performed in triplicate. Selenocystamine (500 mM stock solution) was diluted in Hypure water and added to each experiment in order to obtain the following final concentrations evaluated in this work: 0.0, 0.1, 1.0, 2.0, 5.0, 10.0, 25.0, 100.0 µM. Samples were withdrawn at 0, 8, 24, 48, 72 and 96 h to measure cell concentration, fluorescence (GFP) and luminescence (Gluc). The cultures of the strain expressing αCD22CH23PE40 (immunotoxin) were fed with cystamine (500 mM stock solution, diluted in water) or selenocystamine at 2.0 µM, their samples collected after 48 h and the protein expression evaluated by ELISA.

### Luminescence assay

Coelenterazine (Fisher Scientific, USA), substrate for Gluc, was dissolved in ethanol to obtain a 1 mM stock solution (Shao and Bock 2008). To assay luciferase activity, triplicates of culture samples were centrifuged at 3,000 rpm (2,000*g*) for 10 min and sonicated in lysis buffer (TBST—500 mM Tris, 1.5 M NaCl and 0.1% tween 20) at 15% amplitude for 15 s (two times each sample). The supernatant (soluble protein fraction) was isolated by centrifugation at 13,200 rpm (16,100*g*) for 10 min, and the protein concentration was quantified by LOWRY assay (Lowry et al. 1951) to have the same volume and total protein concentration for each analysis. Immediately before luminescence analysis, a mixture of coelenterazine and buffer [0.1 M K_2_HPO_4_ (pH 7.6), 0.5 M NaCl, 1 mM EDTA] at the ratio 1:50 (volume:volume) was added at the top of the culture by the injector of an Infinite M200 PRO plate reader (Tecan, Männedorf, Switzerland), which added 50 µL of substrate, followed by shaking for 3 s, waiting for 10 s, reading at automatic attenuation, with integration time of 1,000 ms and settle time of 150 ms. Gluc luminescence is linear related to substrate (coelenterazine) concentration between 0.1 and 10 µM (Wille et al. 2012); for this reason, in all experiments, substrate was added in excess to guarantee that all the enzyme was catalyzing the reaction to produce light for all the experiments. All coelenterazine solutions were kept at −20°C, and working solutions were kept on ice and in the dark.

### Fluorescence assay

Triplicate culture samples (100 µL) were transferred directly to wells of a 96-well black plate, which was read using an Infinite M200 PRO plate reader (Tecan, Männedorf, Switzerland). Fluorescence measurements were taken at 488/522 excitation/emission and TAP medium was used as blank. All the results of fluorescence were normalized to cell concentration (cells mL^−1^).

### ELISA assay

ELISA antigen binding assays were performed in 96-well microtiter plates (Costar, Corning, NY, USA), which were coated with 50 µL of 10 ng µL^−1^ of total protein obtained from experiments carried out with the strain expressing αCD22CH23PE40 immunotoxin proteins. Plates were blocked with TBS buffer containing 1% bovine serum albumin (BSA). Wells were washed three times with 250 µL of TBS and primary antibody (ETA—exotoxin A) was added at a concentration of 1:5,000 (antibody:blocking buffer). After a washing step, secondary HRP (horseradish peroxidase) conjugated antibody was applied at the concentration (1:10,000). Peroxide solution (H_2_O_2_) and peroxidase substrate (TMB, Pierce) were premixed and 100 µL of this solution was added to each well, followed by 100 µL of a 2 M H_2_SO_4_ solution to stop the reaction. The plates were visualized at 450 nm and binding was quantified by color using a Spectra Max 250 plate reader.

A calibration curve was carried out for each ELISA plate, by diluting samples of purified αCD22CH23PE40 immunotoxin protein (Tran et al. 2013b) with a control strain, to have four different expression percentages: 0.0, 0.5, 1.0 and 5.0, which corresponds to: 0.0, 0.05, 0.10, 0.5 mg mL^−1^ of purified immunotoxin, respectively.

## Results

### Plasmids constructions and chloroplast transformations

*Chlamydomonas reinhardtii* w1.1 strain was genetically transformed by particle bombardment and the recombinant genes, in transformation vectors containing kanamycin resistance gene, were inserted into the psbA site of the chloroplast genome (Figure 1a). PCR of cell lysates was performed to verify the presence of Gluc or GFP (Figure 1b, c). Subsequently, gene positives were screened for homoplasmicity to guarantee that all copies of the chloroplast genome did not contain the SAA gene that will be replaced (Figure 1d, e).

### Cell growth

A cell growth comparison was made between the two recombinant strains GFP and Gluc. The cell concentration was measured for all the cultivations in the absence or presence of increasing selenocystamine final concentrations (0.0, 0.1, 1.0, 2.0, 5.0, 10.0, 25.0 and 100.0 µM) during 96 h at the time points 0, 8, 24, 48, 72 and 96 h.

**Figure 2:**
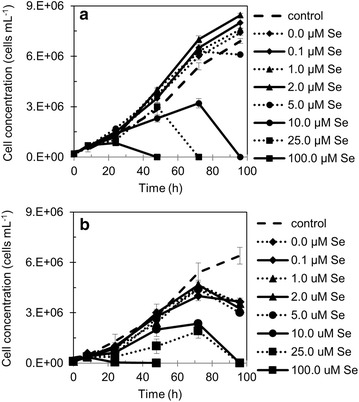
Growth curves of recombinant *Chlamydomonas reinhardtii* strains cultivations treated with increasing concentrations of selenocystamine. **a** Cell concentration (cells mL^−1^) as a function of time (h), for recombinant *C. reinhardtii*—Gluc (strain expressing *Gaussia* luciferase) cultivations containing: 0.0, 0.1, 1.0, 2.0, 5.0, 10.0, 25.0 and 100.0 µM selenocystamine final concentration in the culture medium, compared to control w1.1 (psbA knockout). **b** Cell concentration (cells mL^−1^) as a function of time (h), for recombinant *C. reinhardtii*—GFP (strain expressing green fluorescent protein) cultivations containing: 0.0, 0.1, 1.0, 2.0, 5.0, 10.0, 25.0 and 100.0 µM selenocystamine final concentration in the culture medium, compared to control w1.1 (psbA knockout). *Error bars* were calculated from triplicate average values of three different experiments.

During the experiments 0.1, 1.0 and 2.0 µM selenocystamine concentrations, cell growth remained similar to the control cultivation that had no selenocystamine addition, without a lag phase at the beginning of the cultivation. In both strains, Gluc and GFP were not affected by the addition of 5.0 µM selenocystamine, however in some cases a yellowish color (indicative of unhealthy cells) was observed after 24 h of cultivation, when compared to the lower concentrations of selenocystamine (data not shown). Growth curves of strains expressing Gluc and GFP (Figure 2a, b) for the experiments 10, 25 or 100 µM selenocystamine concentrations showed greatly reduced cell concentration compared to the other experiments. Cell concentration values for those three highest selenocystamine concentration experiments decreased after 24 h, and cells were dead before the end of cultivation.

### Gluc (Gaussia luciferase) luminescence assay

Gluc, strain expressing luciferase from *Gaussia princeps*, was cultivated in the absence or presence of selenocystamine increasing concentrations (0.0, 0.1, 1.0, 2.0, 5.0, 10.0, 25.0 and 100.0 µM). Gluc luminescence was analyzed for every time point and compared to the control strain (w1.1).

**Figure 3:**
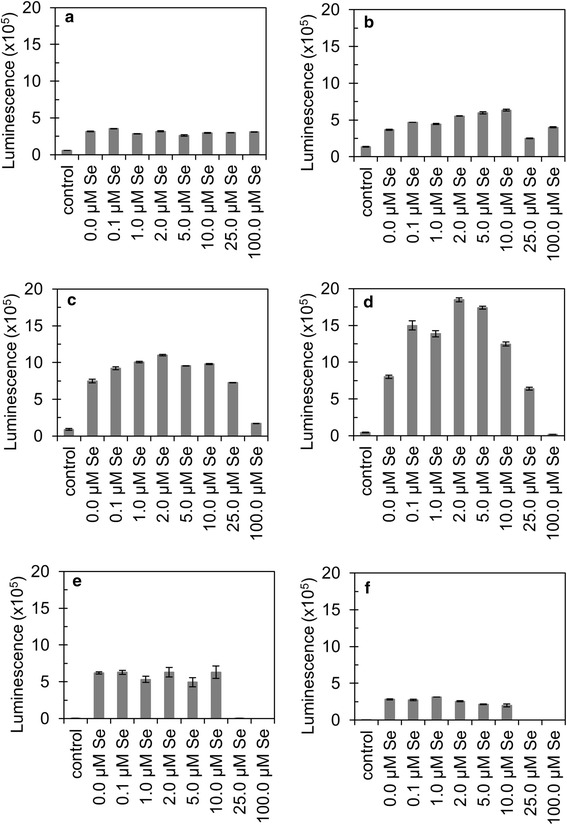
Luminescence of recombinant *C. reinhardtii*—Gluc (strain expressing *Gaussia* luciferase protein) cultivations supplemented with selenocystamine. This oxidative molecule was supplemented to obtain the following final concentrations: 0.0, 0.1, 1.0, 2.0, 5.0, 10.0, 25.0 and 100.0 µM, and these were compared to control w1.1. **a** 0 h of cultivation; **b** 8 h; **c** 24 h; **d** 48 h; **e** 72 h; **f** 96 h. All *error bars* were calculated by using the triplicate average values of different experiments, and the values were equalized to total protein concentration.

At time zero (Figure 3a), as expected, no effect on luminescence was detected in any treatment sample. After 8 h we found that higher selenocystamine concentrations resulted in higher luminescence levels, except in the experiments 25 and 100 µM selenocystamine concentrations, where luminescence appeared to be significantly lower (Figure 3b). After 24 h (Figure 3c), the luminescence value was significantly higher for 2.0 µM selenocystamine compared to the control. At 48 h of cultivation, the luminescence values were the highest compared to the other time points. At 72 and 96 h the luminescence values started to decrease compared to the earlier time points (Figure 3d–f).

### GFP fluorescence assay

The recombinant strain expressing GFP in the chloroplast was cultivated in the absence or presence of selenocystamine increasing concentrations (0.0, 0.1, 1.0, 2.0, 5.0, 10.0, 25.0 and 100.0 µM). GFP relative fluorescence was analyzed for every time point and compared to the control strain (w1.1).

**Figure 4:**
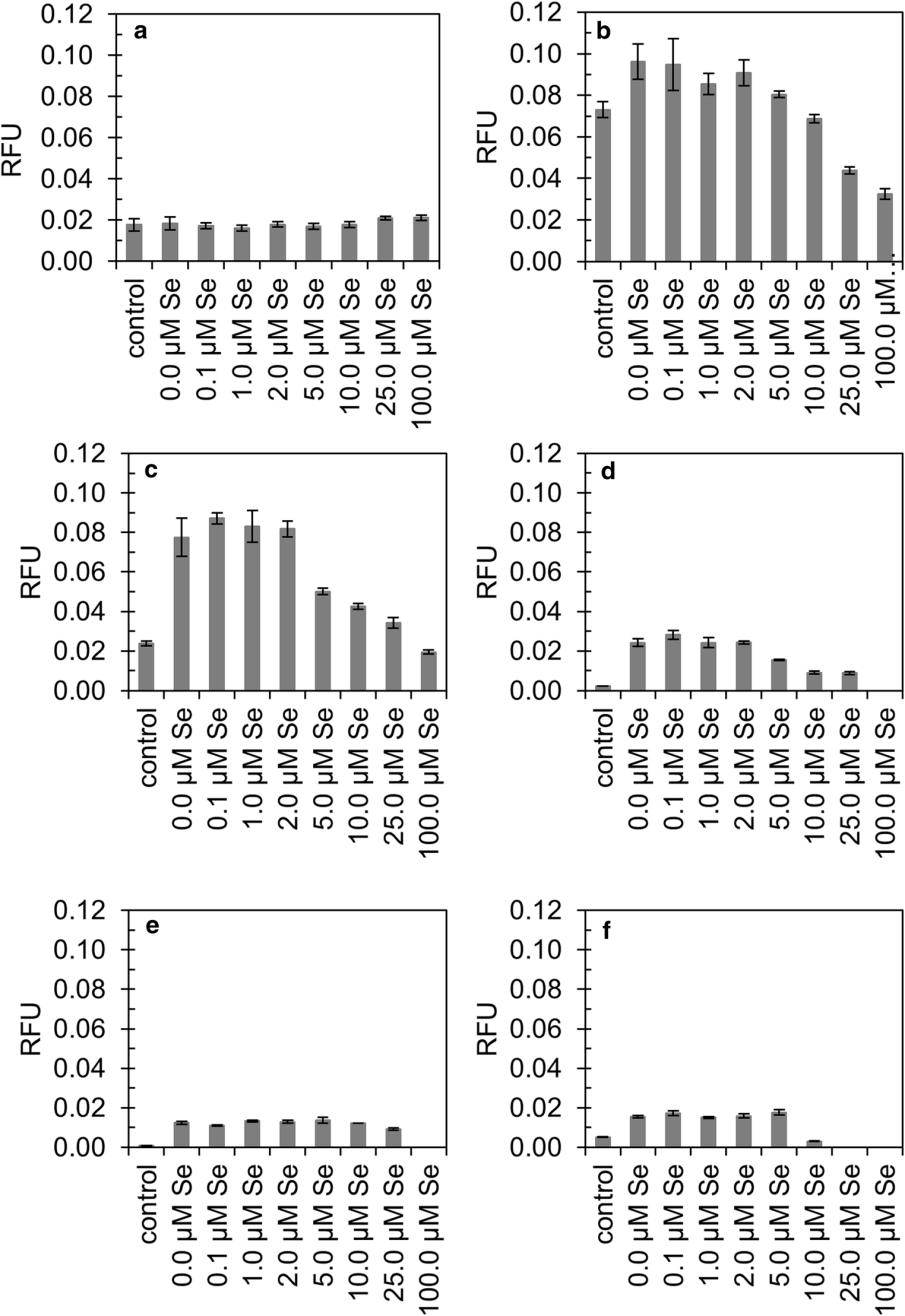
Fluorescence of recombinant *C. reinhardtii*—GFP (strain expressing green fluorescent protein) cultivations supplemented with selenocystamine. This oxidative molecule was supplemented to obtain the following final concentrations: 0.0, 0.1, 1.0, 2.0, 5.0, 10.0, 25.0 and 100.0 µM, and these were compared to control w1.1. **a** 0 h of cultivation; **b** 8 h; **c** 24 h; **d** 48 h; **e** 72 h; **f** 96 h. *RFU* relative fluorescence unit. All *error bars* were calculated by using the triplicate average values, which were also equalized to cell concentration measurements.

The experiments with lower selenocystamine concentrations (0.1, 1.0, 2.0 and 5.0 µM) showed no impact on GFP fluorescence, while high concentrations (10.0, 25.0 and 100.0 µM) showed decreasing fluorescence signal. At time zero (Figure 4a), before flasks were placed under light growth conditions, relative fluorescence unit (RFU) values were the same across all experimental conditions. After 8 h (Figure 4b), a significant decrease in RFU values was observed in experiments with 25.0 and 100.0 µM selenocystamine. After 24 and 48 h of cultivation, 0, 0.1, 1.0 and 2.0 µM selenocystamine additions showed very similar fluorescence values (Figure 4c, d), while in the experiments 5.0, 10.0, 25.0 and 100.0 µM selenocystamine concentrations, lower RFU values were observed. At 72 and 96 h (Figure 4e, f), RFU values were much lower than the other time points and at the same level between the experiments.

### Immunotoxin αCD22CH23PE40 ELISA

**Figure 5:**
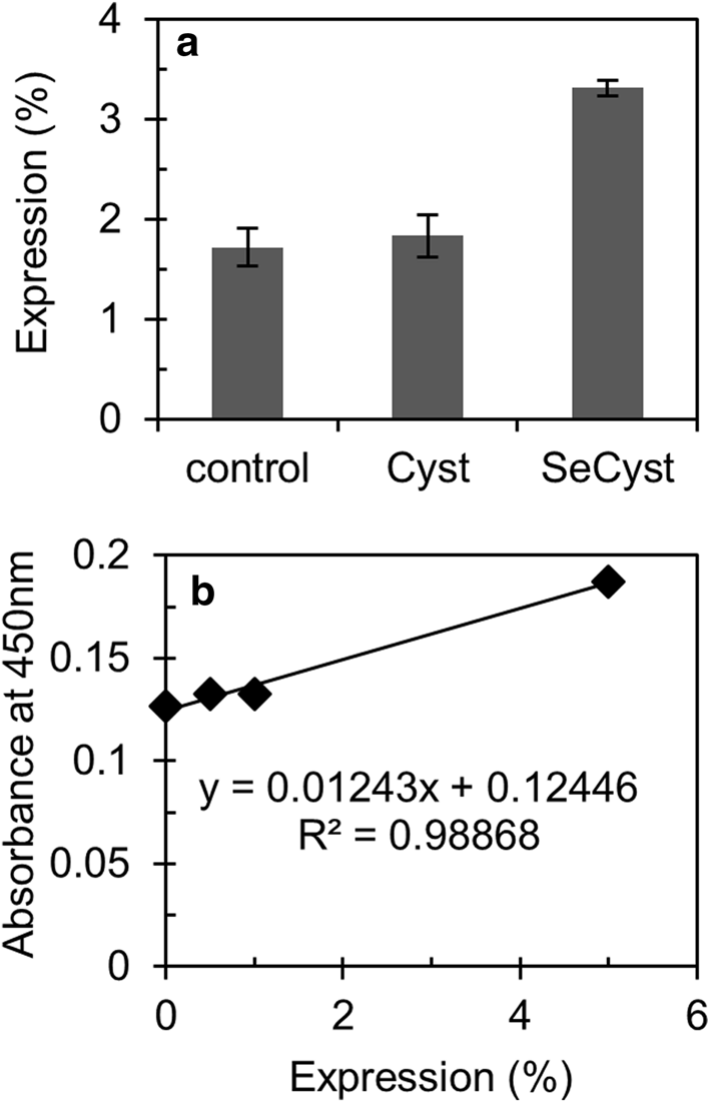
Immunotoxin (αCD22CH23PE40) expression in *Chlamydomonas reinhardtii* chloroplast. **a** The *bar graph* shows the percentage of expression for samples withdrawn after 48 h of cultivation in each experiment, which had 2.0 µM final concentration of cystamine (Cys) or selenocystamine (Secyst) compared to control (w1.1). All *error bars* were calculated by using the triplicate average values, which were also equalized to total protein concentration. **b** Calibration curve used for quantitative ELISA assay. Absorbance is shown as a function of recombinant protein αCD22CH23PE40 expression in percentages (0.0, 0.5, 1.0 and 5.0%).

The recombinant strain expressing the immunotoxin αCD22CH23PE40 was cultivated in the absence or presence of selenocystamine or cystamine at the concentration of 2.0 µM. Samples were taken after 48 h of cultivation, and an ELISA was carried out to compare the accumulation percentage between the diselenide or disulfide supplementation. The results of ELISA indicate that cells accumulated more immunotoxin protein when selenocystamine molecule was added to the cultivation media when compared to cystamine and to the control (Figure 5a). The calibration curve presented in Figure 5b was used to obtain the immunotoxin accumulation percentages and to compare different experiments.

## Discussion

In this work we set out to evaluate the ability of a small molecule oxidant, selenocystamine, to increase protein accumulation of heterologous proteins that require the formation of disulfide bonds in algal chloroplasts. Chloroplasts of *C. reinhardtii* were transformed with genes coding for Gluc (Goerke et al. 2008) that require disulfide bonds to be active, or GFP (Prisco et al. 2005) that does not. Stable strains expressing each of the genes were generated and cultured in the absence or presence of selenocystamine increasing concentrations. Subsequently, their heterologous protein content was quantified to determine if selenocystamine had an effect on protein activity. When analyzed, it was demonstrated that only the strain expressing protein that contains disulfide bonds, Gluc, showed an increase in this heterologous protein. The increase of Gluc luminescence when compared to the control evidences the higher Gluc activity, which is correlated to the increase of disulfide bonds formation (Goerke et al. 2008). In contrast, when selenocystamine was fed to a strain containing GFP, a reporter protein that requires no disulfide bonds for activity, there was no significant change in protein accumulation that was observed. The best concentration of selenocystamine for Gluc activity increase was the chosen one to evaluate the effect of cystamine and selenocystamine, small oxidative molecules, on a stable strain expressing a more complex protein, immunotoxin (Tran et al. 2013b). The strain expressing this complex therapeutic protein, containing 12 disulfide bonds, also showed an increase of the recombinant protein accumulation.

Selenocystamine is a small molecule diselenide known for its ability to increase the rate of oxidative protein folding to allow proteins to achieve their native state (Beld et al. 2010). Here we have presented data that demonstrates selenocystamine has an impact on increase of recombinant protein in algal chloroplast when this protein requires the formation of disulfide bonds for biological activity. Redox potential and the transfer of electrons plays a key role in chloroplast metabolism during photosynthesis (Johnson and Alric 2013) and translation (Trebitsh et al. 2000). During photosynthesis, redox potential is generated through the transfer of an electron through the photosynthetic core apparatus. This redox potential is used to fix carbon dioxide into a storable energy source. Additionally, the reducing potential generated by photosynthesis has been shown to initiate a chain of redox reactions ultimately leading to the reduction of a nuclear-encoded protein, RB60 that initiates translation of genes under the control of the regulatory elements of the psbA 5′-UTR. With these pivotal roles that redox plays it could be possible for selenocystamine to have a universal impact on protein accumulation through processes such as redox regulated translational activation or a limited role by stabilizing proteins that require disulfide bonds (Kim and Mayfield 1997). No increases were seen in GFP accumulation, suggesting that selenocystamine plays a role in stabilizing protein structures through the formation of disulfide bonds and potentially mitigating degradation of un-folded polypeptides. Although selenocystamine was able to increase protein accumulation, at concentrations greater than 5 µM, it became toxic to *C. reinhardtii* cultures. A similar toxicity was observed in *Saccharomyces cerevisiae* (Beld 2009). This toxicity is not unique to selenocystamine but appears to be a general effect of oxidizing molecules such as glutathione or 5,5′-dithiobis[2-nitrobenzoic acid] (Wakabayashi and King 2006) which caused *C. reinhardtii* cells to lose motility. Interestingly, when tested in *S. cerevisiae*, selenocystamine did not significantly increase protein accumulation of proteins containing disulfide bonds. This data suggest that *C. reinhardtii* is more efficient in selenocystamine uptake into its chloroplast than *S. cerevisiae* cultures. It should also be noted that concentrations of selenocystamine that were able to increase recombinant active protein containing disulfide bonds in *C. reinhardtii* chloroplasts were similar to the concentrations that were observed to impact protein accumulation in *E. coli* cultures (Beld et al. 2010).

Increasing the number of disulfide bonds in a protein generally increases the difficulty for a cell to achieve a stable conformation of the protein product. This inefficiency will lead to degraded protein and an overall decrease in accumulation. To determine if cystamine or selenocystamine could impact the accumulation of a protein that has potential therapeutic relevance, these molecules were added to *C. reinhardtii* cultures expressing an immunotoxin with 12 disulfide bonds. Immunotoxin expressed in *C. reinhardtii* chloroplasts by Tran et al. (2013b), binds target B-cells and kills them, making these proteins potential cancer therapies. Using the concentration of selenocystamine that had the greatest impact on Gluc accumulation (2.0 µM), we fed both oxidative molecules (cystamine or selenocystamine) to cultures expressing the immunotoxin (αCD22HCH23PE40) and detected a doubling of accumulation of immunotoxin proteins, when selenocystamine was fed. These result could be explained by the fact that diselenides (selenocystamine) are much stronger electrophile than disulfides (cystamine), and also, the rate of selenol/diselenide exchange is much faster than the corresponding rate of thiol/disulfide exchange (Hondal et al. 2013; Pleasants et al. 1989). Thus, the action of selenocystamine, unlike cystamine, shows to positively impact protein accumulation in proteins that require the formation of many disulfide bonds to achieve a stable conformation.

Our results demonstrate that a small molecule diselenide can improve the heterologous accumulation and activity of proteins containing disulfide bonds in *C. reinhardtii* chloroplast, whereas proteins without disulfide bonds shows no apparent increase in yield. These findings imply that limitations in oxidative protein folding are indeed partially responsible for lower yields of proteins containing disulfide bonds like the immunotoxin. Similar to protein expression in bacteria, there is a clearly need for engineered algal strains with optimized oxidative protein folding. Strategies such as the introduction of proteins that assist with oxidative protein folding, such as chaperones and PDIs, could dramatically increase the yields of these complex proteins in a scalable manner. Overall, algal protein expression is coming of age, and with it the tools and understanding required to develop an optimal algal strain for heterologous protein expression.

## References

[CR1] Ahmad M, Hirz M, Pichler H, Schwab H (2014). Protein expression in *Pichia pastoris*: recent achievements and perspectives for heterologous protein production. Appl Microbiol Biotechnol.

[CR2] Barrera DJ, Rosenberg JN, Chiu JG, Chang YN, Debatis M, Ngoi SM (2014). Algal chloroplast produced camelid V H antitoxins are capable of neutralizing botulinum neurotoxin. Plant Biotechnol J.

[CR100] Beld J (2009) Small molecule diselenides as probes of oxidative protein folding. Dissertation, Eidgenössische Technische Hochschule ETH Zürich. http://dx.doi.org/10.3929/ethz-a-005950993

[CR3] Beld J, Woycechowsky KJ, Hilvert D (2007). Selenoglutathione: efficient oxidative protein folding by a diselenide. Biochemistry.

[CR4] Beld J, Woycechowsky KJ, Hilvert D (2010). Small-molecule diselenides catalyze oxidative protein folding in vivo. ACS Chem Biol.

[CR5] Boynton JE, Gillham NW, Harris EH, Hosler JP, Johnson AM, Jones AR (1988). Chloroplast transformation in *Chlamydomonas* with high velocity microprojectiles. Science.

[CR6] Breiman A, Fawcett TW, Ghirardi ML, Mattoo AK (1992). Plant organelles contain distinct peptidylprolyl cis, trans-isomerases. J Biol Chem.

[CR7] Franklin SE, Mayfield SP (2004). Prospects for molecular farming in the green alga *Chlamydomonas*. Curr Opin Plant Biol.

[CR8] Goerke AR, Loening AM, Gambhir SS, Swartz JR (2008). Cell-free metabolic engineering promotes high-level production of bioactive *Gaussia princeps* luciferase. Metab Eng.

[CR9] Gorman DS, Levine RP (1965). Cytochrome f and plastocyanin: their sequence in the photosynthetic electron transport chain of *Chlamydomonas reinhardi*. Proc Natl Acad Sci USA.

[CR10] Gregory JA, Li F, Tomosada LM, Cox CJ, Topol AB, Vinetz JM (2012). Algae-produced Pfs25 elicits antibodies that inhibit malaria transmission. PLoS One.

[CR11] Grossman AR (2000). *Chlamydomonas reinhardtii* and photosynthesis: genetics to genomics. Curr Opin Plant Biol.

[CR12] Hondal RJ, Marino SM, Gladyshev VN (2013). Selenocysteine in thiol/disulfide-like exchange reactions. Antioxid Redox Signal.

[CR13] Horwich AL, Weber-Ban EU, Finley D (1999). Chaperone rings in protein folding and degradation. P Natl Acad Sci USA.

[CR14] Johnson IS (1983). Human insulin from recombinant DNA technology. Science.

[CR15] Johnson X, Alric J (2013). Central carbon metabolism and electron transport in *Chlamydomonas reinhardtii*: metabolic constraints for carbon partitioning between oil and starch. Eukaryot Cell.

[CR16] Kim J, Mayfield SP (1997). Protein disulfide isomerase as a regulator of chloroplast translational activation. Science.

[CR17] Kim JY, Kim YG, Lee GM (2012). CHO cells in biotechnology for production of recombinant proteins: current state and further potential. Appl Microbiol Biotechnol.

[CR18] Krief A, Trabelsi M, Dumont W, Derock M (2004). Conditions-driven selective synthesis of selenides and selenols from elemental selenium. Synlett.

[CR19] Levitan A, Trebitsh T, Kiss V, Pereg Y, Dangoor I, Danon A (2005). Dual targeting of the protein disulfide isomerase RB60 to the chloroplast and the endoplasmic reticulum. Proc Natl Acad Sci USA.

[CR20] Lilie H, Mclaughlin S, Freedman R, Buchner J (1994). Influence of protein disulfide-isomerase (Pdi) on antibody folding in-vitro. J Biol Chem.

[CR21] Lowry OH, Rosebrough NJ, Farr AL, Randall RJ (1951). Protein measurement with the Folin phenol reagent. J Biol Chem.

[CR22] Manuell AL, Beligni MV, Elder JH, Siefker DT, Tran M, Weber A (2007). Robust expression of a bioactive mammalian protein in *Chlamydomonas* chloroplast. Plant Biotechnol J.

[CR23] Nakamura Y, Gojobori T, Ikemura T (1999). Codon usage tabulated from the international DNA sequence databases; its status 1999. Nucleic Acids Res.

[CR24] Nauser T, Steinmann D, Koppenol WH (2012). Why do proteins use selenocysteine instead of cysteine?. Amino Acids.

[CR25] Pleasants JC, Guo W, Rabenstein DL (1989). A comparative study of the kinetics of selenol/diselenide and thiol/disulfide exchange reactions. J Am Chem Soc.

[CR26] Popescu CE, Lee RW (2007). Mitochondrial genome sequence evolution in *Chlamydomonas*. Genetics.

[CR27] Prisco U, Leung C, Xirouchaki C, Jones CH, Heath JK, Palmer RE (2005). Residue-specific immobilization of protein molecules by size-selected clusters. J R Soc Interface.

[CR28] Rasala BA, Lee PA, Shen Z, Briggs SP, Mendez M, Mayfield SP (2012). Robust expression and secretion of Xylanase1 in *Chlamydomonas reinhardtii* by fusion to a selection gene and processing with the FMDV 2A peptide. PLoS One.

[CR29] Rosano GL, Ceccarelli EA (2014). Recombinant protein expression in *Escherichia coli*: advances and challenges. Front Microbiol.

[CR30] Sato Y, Inaba K (2012). Disulfide bond formation network in the three biological kingdoms, bacteria, fungi and mammals. FEBS J.

[CR31] Schroda M (2004). The *Chlamydomonas* genome reveals its secrets: chaperone genes and the potential roles of their gene products in the chloroplast. Photosynth Res.

[CR32] Schroder M (2008). Engineering eukaryotic protein factories. Biotechnol Lett.

[CR33] Shao N, Bock R (2008). A codon-optimized luciferase from Gaussia princeps facilitates the in vivo monitoring of gene expression in the model alga *Chlamydomonas reinhardtii*. Curr Genet.

[CR34] Specht E, Miyake-Stoner S, Mayfield S (2010). Micro-algae come of age as a platform for recombinant protein production. Biotechnol Lett.

[CR35] Tran M, Mayfield SP (2010) Expression of full length monoclonal antibodies (mAb) in algal chloroplast. In: Antibody engineering, vol. 1, 2nd edn, pp 503–516. doi:10.1007/978-3-642-01144-3_32

[CR36] Tran M, Zhou B, Pettersson PL, Gonzalez MJ, Mayfield SP (2009). Synthesis and assembly of a full-length human monoclonal antibody in algal chloroplasts. Biotechnol Bioeng.

[CR37] Tran M, Henry RE, Siefker D, Van C, Newkirk G, Kim J (2013). Production of anti-cancer immunotoxins in algae: ribosome inactivating proteins as fusion partners. Biotechnol Bioeng.

[CR38] Tran M, Van C, Barrera DJ, Pettersson PL, Peinado CD, Bui J (2013). Production of unique immunotoxin cancer therapeutics in algal chloroplasts. Proc Natl Acad Sci USA.

[CR39] Trebitsh T, Levitan A, Sofer A, Danon A (2000). Translation of chloroplast psbA mRNA is modulated in the light by counteracting oxidizing and reducing activities. Mol Cell Biol.

[CR40] Vrljic M, Strop P, Hill RC, Hansen KC, Chu S, Brunger AT (2011). Post-translational modifications and lipid binding profile of insect cell-expressed full-length mammalian synaptotagmin 1. Biochemistry.

[CR41] Wakabayashi K, King SM (2006). Modulation of *Chlamydomonas reinhardtii* flagellar motility by redox poise. J Cell Biol.

[CR42] Walsh G (2014). Biopharmaceutical benchmarks 2014. Nat Biotechnol.

[CR43] Wilkinson B, Gilbert HF (2004). Protein disulfide isomerase. Biochim Biophys Acta.

[CR44] Wille T, Blank K, Schmidt C, Vogt V, Gerlach RG (2012). *Gaussia princeps* luciferase as a reporter for transcriptional activity, protein secretion, and protein-protein interactions in *Salmonella enterica* s*erovar typhimurium*. Appl Environ Microbiol.

